# Metabolic and transcriptional analyses in response to potent inhibitors establish MEP pathway as major route for camptothecin biosynthesis in *Nothapodytes nimmoniana* (Graham) Mabb

**DOI:** 10.1186/s12870-019-1912-x

**Published:** 2019-07-10

**Authors:** Gulzar A. Rather, Arti Sharma, Syed Mudassir Jeelani, Prashant Misra, Veenu Kaul, Surrinder K. Lattoo

**Affiliations:** 1 Plant Biotechnology Division, CSIR- Indian Institute of Integrative Medicine, Canal Road, Jammu Tawi, 180001 India; 2 Department of Botany, University of Jammu, Jammu Tawi, 180006 India

**Keywords:** Camptothecin, Fosmidomycin, Lovastatin, MEP and MVA pathway, *Nothapodytes nimmoniana*, Shoot culture, Transformed hairy roots

## Abstract

**Background:**

*Nothapodytes nimmoniana*, a plant of pivotal medicinal significance is a source of potent anticancer monoterpene indole alkaloid (MIA) camptothecin (CPT). This compound owes its potency due to topoisomerase-I inhibitory activity. However, biosynthetic and regulatory aspects of CPT biosynthesis so far remain elusive. Production of CPT is also constrained due to unavailability of suitable in vitro experimental system. Contextually, there are two routes for the biosynthesis of MIAs: the mevalonate (MVA) pathway operating in cytosol and the methylerythritol phosphate (MEP) pathway in the plastids. Determination of relative precursor flux through either of these pathways may provide a new vista for manipulating the enhanced CPT production.

**Results:**

In present study, specific enzyme inhibitors of MVA (lovastatin) and MEP pathways (fosmidomycin) were used to perturb the metabolic flux in *N. nimmoniana.* Interaction of both these pathways was investigated at transcriptional level by using qRT-PCR and at metabolite level by evaluating secologanin, tryptamine and CPT contents. In fosmidomycin treated plants, highly significant reduction was observed in both secologanin and CPT accumulation in the range 40–57% and 64–71.5% respectively, while 4.61–7.69% increase was observed in tryptamine content as compared to control. Lovastatin treatment showed reduction in CPT (7–11%) and secologanin (7.5%) accumulation while tryptamine registered slight increase (3.84%) in comparison to control. These inhibitor mediated changes were reflected at transcriptional level via altering expression levels of deoxy-xylulose-5-phosphate reductoisomerase (DXR) and hydroxymethylglutaryl-CoA reductase (HMG). Further, mRNA expression of four more genes downstream to DXR and HMG of MEP and MVA pathways respectively were also investigated. Expression analysis also included secologanin synthase (SLS) and strictosidine synthase (STR) of seco-iridoid pathway. Present investigation also entailed development of an efficient in vitro multiplication system as a precursor to pathway flux studies. Further, a robust *Agrobacterium-*mediated transformed hairy root protocol was also developed for its amenability for up-scaling as a future prospect.

**Conclusions:**

Metabolic and transcriptional changes reveal differential efficacy of cytosolic and plastidial inhibitors in context to pathway flux perturbations on seco-iridoid end-product camptothecin. MEP pathway plausibly is the major precursor contributor towards CPT production. These empirical findings allude towards developing suitable biotechnological interventions for enhanced CPT production.

**Electronic supplementary material:**

The online version of this article (10.1186/s12870-019-1912-x) contains supplementary material, which is available to authorized users.

## Background

*Nothapodytes nimmoniana* (Graham) Mabb. (Icacinaceae), a highly valued medicinal plant is distributed in Western Ghats and North-eastern India and in Myanmar, Sri Lanka and Thailand. The species is one of the richest sources of pharmacologically active monoterpene indole alkaloid (MIA), camptothecin (0.150–2.620%) [1]. The other important sources of CPT include *Camptotheca accuminata* (0.24–0.5%), *Merriliodendron megacarpum* (0.053%), *Ophiorrhiza pumila (0.1%)*, *Ervatamia heyneana (0.13%)*, *Mostuea brunonis(0.06%)* and *Pyrenacantha volubilis* (1.35%) [2–4]. CPT is a cytotoxic drug and acts as a strong inhibitor of DNA topoisomerase-I. Its semi-synthetic soluble analogs irinotecan and topotecan exhibit excellent pharmacological properties and clinical efficacy in comparison to CPT [5]. Both irinotecan and topotecan are US-FDA approved (United States, Food and Drug Administration) drugs used for the treatment of colorectal, ovarian, lung and breast cancers [6]. One more novel CPT derivative (7-N-[(substituted-sulfonyl) piperazinyl]-methyl) has been synthesized by incorporation of sulfonylpiperazinyl motif into position-7 of CPT that is effective against five types of human tumour cell lines viz. A-549, KB, MDA-MB-231, MCF-7 and KB-VIN [7]. Other derivatives such as rubitecan, exatecan, 9-nitrocamptothecin, 9-aminocamptothecin and FL118 are currently under clinical investigations [8]. CPT is structurally too complex and its production by chemical synthesis so far has remained elusive and prohibitive. However, extraction of CPT from its natural resources seems difficult to meet its ever increasing market demand [9]. The production of semi-synthetic derivatives relies entirely on CPT and this has subjected *N. nimmoniana* to tremendous anthropogenic pressure in the natural stands [10–12]. Recently, the worldwide market of CPT derivatives has reached around 4 billion US dollars and its ever increasing demand necessitates sustained exploration of the natural resources and alternative production platforms [13]. In this context, in vitro strategies for pathway intensification/manipulation are emerging as an attractive tool to produce high-value secondary metabolites due to various advantages. These mainly include rapid growth rate, genetic stability and their amenability to scale-up to bioreactor level. To circumvent the low yield of biologically active compounds, development and application of genetic engineering tools provide a promising approach to enhance secondary metabolite yields by introducing multiple pathway genes into homologous/heterologous hosts, followed by culturing of transgenic lines on a large scale [14]. *Agrobacterium rhizogenes* mediated hairy root system is more feasible, fast growing and commercially an applicable way to produce higher quantities of desired phytochemicals by over-expressing key pathway genes. Over-expression of ORCA2 and ORCA4 transcription factors in hairy roots of *Catharanthus roseus* boost up 2–40 fold expression levels of both indole and seco-iridoid pathway genes which resulted into higher accumulation of tabersonine, ajmalicine and catharanthine as compared to control [15, 16].

**Fig. 1:**
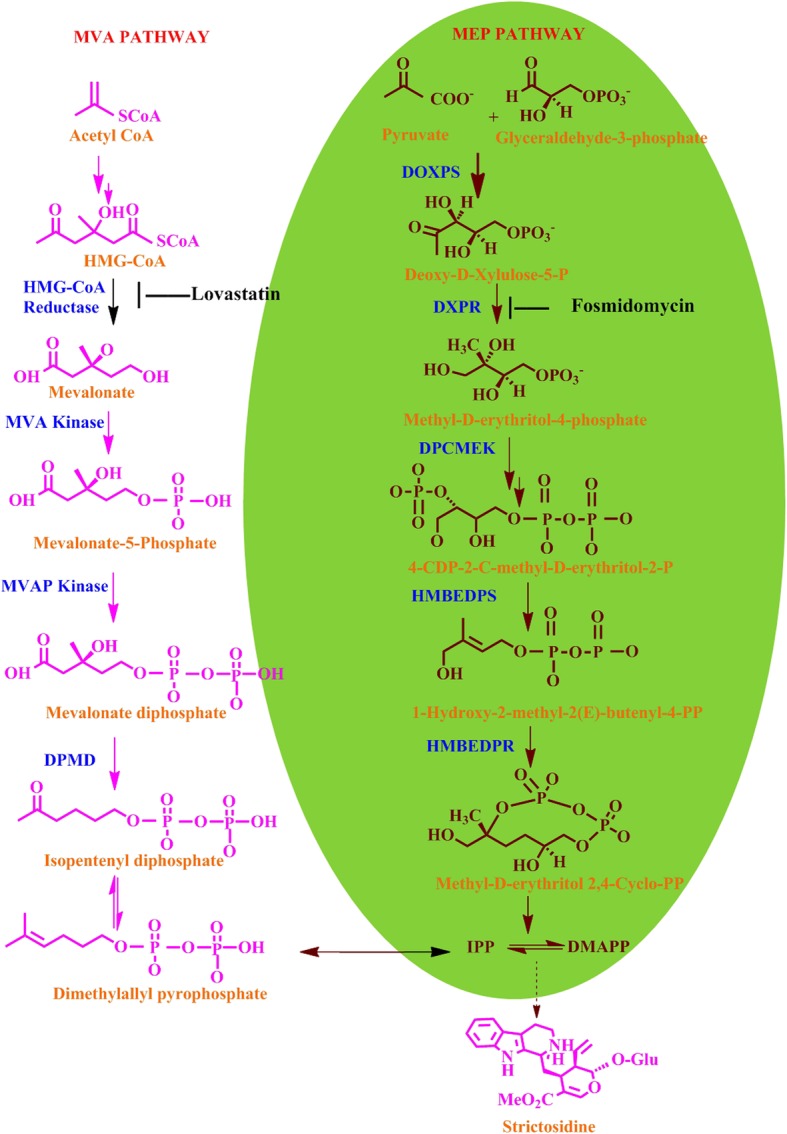
An overview of mevalonate and non-mevalonate pathways: DXR- deoxy-xylulose-5-phosphate reductoisomerase; MEPCT-methyl erythritol-4-phosphate cytidyltransferase; DPCMEK-diphosphocytidyl-2-methyl-D-erythritol kinase; MECDPS-methyl-D-erythritol 2,4-cyclodiphosphate synthase; HMBEDPR-hydroxy-3-methyl but-2-enyl diphosphate reductase; HMG; Hydroxymethylglutaryl-CoA reductase; MK-mevalonate kinase;PMK-phosphomevalonate kinase; DPMD-diphosphomevalonate decarboxylase

The prevailing concept regarding the production of MIAs involves two distinct pathways: the MEP pathway existing in plastids and MVA pathway in the cytosol. The plastidial MEP pathway involving condensation of pyruvate and glyceraldehyde-3-phosphate in presence of 1-deoxy-D-xylulose-5-phosphate synthase resulted in 1-deoxy-D-xylulose 5-phosphate, a first intermediate involved for the synthesis of phytohormones, isoprene, carotenoids, plastoquinone and side chains of chlorophylls. The cytosolic MVA pathway starting from acetyl-CoA and proceeds via mevalonate intermediate, provides the precursors for polyprenols, ubiquinone and sterols [17, 18]. Similarly, 3-Hydroxy-3-methylglutaryl CoA reductase (HMGR) is a crucial enzyme of the MVA pathway and lovastatin (Lov) is a potent inhibitor of HMGR [19]. The 1-deoxy-D-xylulose 5-phosphate reductoisomerase (DXR) is an important enzyme of the MEP pathway, and fosmidomycin (FOS) is its specific inhibitor [20]. These two distinct biosynthetic routes produce dimethylallyl diphosphate (DMAPP) and its isomer isopentenyl pyrophosphate (IPP) that provide a strong flux for the production secologanin a key intermediate of seco-iridoid pathway [21, 22]. Secologanin further condenses with shikimate pathway derivative tryptamine to yield strictosidine which is a universal precursor of all MIAs (Fig. 1) [23–26]. Although, sub-cellular compartmentalization of MEP and MVA pathways allows them to operate simultaneously in plants but the exchange of metabolites between the pathways may occur as indicated in some recent investigations [20, 27]. From previous studies, contribution of both these pathways has been reported in the biosynthesis of some secondary metabolites like homoterpene- 4,8-dimethylnona-1,3,7-triene (DMNT). It is elicited in many higher plants in response to herbivore attack [28]. Moreover, MEP has been reported as a major route for production of seco-iridoid moiety secologanin in *C. roseus* [29] and taxol in *Taxus baccata* [30]. Similarly, in *Picrorhiza kurroa*, by using different enzyme inhibitors (fosmidomycin, mevinolin, glyphosate and aminooxy acetic acid) and transcriptional inhibitor actinomycin D, confirmed that non-mevalonate pathway is the main source of picroside biosynthesis [31]. In *Arabidopsis*, transient reduction of sterol levels were observed in presence of HMGR inhibitor (lovastatin), indicating that the plastidial MEP pathway compensates for the lack of cytosolic IPP required for synthesis of cytosolic sterols [32]. But in case of *Lithospermum erythrorhizon* and *Arnebia euchroma* inhibition studies have shown that shikonin a hemiterpene is produced via MVA pathway which is in contrast to all other mono- and diterpene formation in plants [33, 34]. In general, secondary metabolite biosynthesis occurs through complex cross-talking among metabolic networks and it is of considerable significance to identify the main route of each and every pathway towards their final product. Inhibitor mediated studies of metabolic pathways can provide significant clues in deciphering a route of target metabolite(s) [32, 35]. However, no such investigations have been undertaken in *N. nimmoniana* to understand the relative contribution of either MVA or MEP pathway in the production of CPT*.* Present investigations undertaken shall provide a rational platform and incisive tool for manipulating the diversion of precursor pool toward desired biosynthetic branch through molecular interventions. Furthermore, this study is in continuation to our recent endeavour related to development of comprehensive transcriptome resource of *N. nimmoniana* to identify putative pathway genes, cytochromes related to CPT biosynthesis and transcription factors. It also entailed artificial microRNA (aMIR) mediated suppression of *Nn*CYP76B6 for suggesting its role in CPT biosynthesis [36]. Tissue-specific chemoprofiling revealed secologanin as a central intermediate of seco-iridoid pathway in *N. nimmoniana* in contrast to secologanic acid in *C. accuminata* [36, 37].

Given the pharmacological potency of CPT and its ever increasing demand, the present study deciphers the relative role of plastidial-MEP and cytoclic-MVA pathways in contributing the precursors toward CPT biosynthesis. From this standpoint, an efficient in vitro regeneration system was established and subsequently deployed to investigate the efficacy of two known inhibitors namely, fosmidomycin and lovastatin of DXR and HMG enzymes regulating the MEP and MVA pathways, respectively. The chemo-profiling of inhibitor treated in vitro cultures revealed drastic reduction of CPT, secologanin accumulation in presence of fosmidomycin. These results suggest the pivotal role of MEP pathway in CPT biosynthesis. Further, the effect of inhibitors was also evaluated at transcriptional level by investigating downstream genes of MEP, MVA and seco-iridoid pathways. There was discernible decrease in the relative transcript levels of genes as compared to control. Additionally, transformed hairy root induction protocol was also established using *Agrobacterium rhizogenes.* As a future prospect, transformed hairy root cultures are being up-scaled for further MVA pathway intensification via molecular interventions.

## Methods

### Plant material and establishment of aseptic cultures

The source plant material was identified and authenticated by Dr. Bikarma Singh, Scientist CSIR- Indian Institute of Integrative Medicine ((32° 44′ N longitude, 74° 55′ E latitude; 305 m in altitude), Jammu, India. A voucher specimen (Accession No. 23002; Name of collector: Dr. Surrinder K. Lattoo; Place of collection: CSIR-IIIM experimental field, Jammu) has been deposited at Janaki Ammal Herbarium (RRLH), CSIR-IIIM, Jammu, India. In vitro cultures were established from the nodal segments of field grown juvenile plants of *Nothapodytes nimmoniana.* The nodal segments of 1–3 cm long having axillary buds were used for initiation of in vitro aseptic cultures. Initially, explants were carefully washed with running tap water and immersed in 1% (v/v) Tween 20 (HiMedia, Mumbai, India) for 15–20 min and then again washed under tap water for 45 min. Further, explants were transferred to 0.1% (w/v) HgCl_2_ (HiMedia, Mumbai, India) for a minute then again rinsed with autoclaved distilled water for 2–3 times under laminar hood. Sterilized segments were transferred in Woody Plant Medium (WPM) [38] supplemented with 3% (w/v) sucrose, 0.7% (w/v) agar (Amresco, Mumbai, India), 1 mg/L IBA and various concentrations of different cytokinins (TDZ, IBA, Kn) as shown in Table 1. Triplicated treatments consisting 25 explants were used for the initiation of in vitro cultures from nodal segments of *N. nimmoniana.* Subsequent transfer of 45 in vitro regenerated shoots per treatment were used for root initiation. Statistical analyses of data were carried out as per Gomez and Gomez [39]. Data was analysed using ANOVA with Duncan’s multiple range test at 5% level of significance. Mean and standard errors of means were calculated. The cultures were maintained at temperature of 22 ± 2 °C with 16 h photoperiod. The light intensity of culture room was set at 25–30 μE/m^2^/s facilitated with 40 W cool white fluorescent lamps (Philips, India). For rooting, in vitro regenerated shoots were transferred to WPM which was fortified with different concentrations of either IAA or IBA.

**Table 1:** Effect of different phytohormone concentrations on shoot regeneration of *Nothapodytes nimmoniana* on Woody Plant Medium (WPM) fortified with different concentrations of kinetin (Kn), thidiazuron (TDZ) and benzylaminopurine (BAP)

Woody Medium + growth regulator			Percentage of explants producing shoots	Average number of shoots per explants^*^
Kn	TDZ	BAP		
4.64 × 10^−6^ M	–	–	43	4.25 ± 0.62^f^
9.29 × 10^− 6^ M	–	–	50	6.5 ± 0.64^d^
13.93 × 10^− 6^ M	–	–	56	6.33 ± 0.61^d^
18.58 × 10^− 6^ M	–	–	47	5.2 ± 0.37^e^
–	4.54 × 10^− 6^ M	–	60	8.7 ± 0.85^c^
–	9.08 × 10^− 6^ M	–	65	9.8 ± 0.60^a^
–	13.62 × 10^− 6^ M	–	63	8.8 ± 0.58^b,c^
**–**	**–**	4.439 × 10^− 6^ M	45	6.25 ± 0.75^d^
–	–	8.879 × 10^− 6^ M	52	7.66 ± 0.55^c,d^
–	–	13.319 × 10^− 6^ M	61	8.2 ± 0.70^b,c^
–	–	17.758 × 10^− 6^ M	54	8.5 ± 0.64^b,c^

^*^Values followed by same letters are not significantly different (*P* ≤ 0.05) as per Duncan’s multiple range test

### Inhibitor treatment

Two compounds fosmidomycin (3-[formyl(hydroxy)amino]propylphosphonic acid) and lovastatin ([(1S,3R,7S,8S,8aR)-8-{2-[(4R,6R)-4-Hydroxy-2-oxo-2H-pyran-6-yl]etyl} -3, 7-dimetyl-1, 2, 3, 7, 8,a hexahydronaphtyl (S)-2-metylbutyrat), known inhibitors of MEP and MAV pathways were procured from Sigma-Aldrich, USA. The stock solutions of fosmidomycin was prepared in autoclaved distilled water and lovastatin was dissolved in ethanol. Both were filter sterilized using 0.22 μm sterile filters (Millipore, Bedford, USA). Filter sterilized fosmidomycin and lovastatin were added separately to autoclaved liquid WPM to a final concentration of 150 μM and 100 μM respectively. In vitro rooted cultures acclimatized in liquid medium for 2 weeks were transferred to 250 mL flasks containing inhibitors and one in basal minimal medium as a control. Samples were harvested at day10 and day 20 of the treatment for quantification of CPT, secologanin, tryptamine and also for qRT-PCR analysis.

### RNA extraction and cDNA synthesis

Total RNA was extracted by using SV Total RNA Isolation System (Promega, Madison, USA) as per manufacturer’s instructions. Concentration of total RNAs was estimated by spectrophotometer (AstraAuriga, Cambridge, UK). Quality of RNA was measured by estimating the ratio of absorbance at 260/280 nm and further checking on formaldehyde denatured agarose gel electrophoresis. cDNA was synthesized by using RevertAid First Strand cDNA Synthesis Kit (Thermoscientific, Vilnius, Lithuania) in a total reaction volume of 20 ml containing 1 μg RNA, 10 mM dNTPs, 10 mM oligo (dT) primer, 1 μl M-MuLV reverse transcriptase (200 U/ml) and 4 μl of 5X first strand buffer (250 mM Tris- HCl, pH 8.3, 250 mM KCl, 20 mM MgCl_2_, 50 mM DTT). The reaction was incubated at 42 °C for 60 min, followed by inactivate of reverse transcriptase at 70 °C for 5 min.

### Sample preparation and HPLC conditions

Extraction of CPT, secologanin and tryptamine from *N. nimmoniana* was performed using a slightly modified protocol as described by Fulzele et al. (2005). Briefly, the plant samples were collected at 10th and 20th day of treatment and were dried at temperature 25 ± 2 °C and relative humidity 65 ± 5%. Dried samples were separately grounded into fine powder using mortar and pestle. The fine powder was extracted with 90% methanol (v/v) by stirring on magnetic stirrer (Multispin motorless stirrer, Tarsons, India) at room temperature (26 ± 2 °C) for 48 h. The extracts were filtered and concentrated using rotary evaporator. The authentic standard camptothecin (1 mg/mL) and the sample extracts (20 mg/mL) were prepared in HPLC grade methanol, filtered through 0.25 μm membrane filters (Millipore, Bedford, USA) and subjected to HPLC analysis. The HPLC analysis was carried out with Shimadzu CLASS-VP V 6.14 SPI system (Tokyo, Japan) equipped with 5 μm, 4.6× 250 mm RP-18e column (E-Merck, Bangalore, India), a quaternary gradient pump (LC-10AT VP model) and PDA detector (Model: SPD-M10A VP). The mobile phase of CPT and secologanin consisted of acetonitrile: formic acid (99.5:0.5; v/v) delivered at flow rate of 800 μL min^− 1^. For determination of tryptamine, mobile phase consisted of 50% (v/v) methanol in water containing 0.1% formic acid delivered at a flow rate of 0.9 mL min^− 1^. The injection volume of the samples was 10 μl and the column temperature was maintained at 30 °C to provide efficiency to the peaks. The UV wavelength was set at 254 nm for detection of chromatograms. Identification and quantification of CPT, secologanin and tryptamine was carried out using a reverse-phase HPLC system. The detection was made on the basis of retention time of standards under specific column conditions.

### LC-MS/MS analysis

Liquid chromatographic tandem mass spectrometry (LC-MS/MS) analysis was performed for the determination of fosmidomycin and lovastatin in the sample extracts of treated plants. LC-MS/MS analysis was carried on on LiChrospher® RP-18 (4.6 × 250 mm inner diameter, 5 μm) column using mobile phase composed of 0.1% (v/v) formic acid in water and 0.2% (v/v) acetic acid in methanol. The flow rate was set at 0.5 mL/min and column temperature was set at 30 °C. Triple quadrupole tandem mass spectrometer was operated using electro spray ionization source in positive ion mode. Other common MS conditions were as follows: DL temperature 225 °C, nebulizer gas flow 3 L/min and drying gas flow were 15 L/min. Total run time was 30 min. Five microliter of sample volume was injected onto LC-MS/MS system. Lab solution software was used to analyse the data.

### qRT-PCR assessment

Gene specific primers for qRT-PCR analysis were designed from the transcriptome resource of *N. nimmoniana* established by the authors [36]. For proper selection, contigs having maximum homology with functionally characterized genes in NCBI were selected after BLASTx search. Primers were designed via Primer Express tool Version 3.0 (Table 2). The total RNA isolated from leaves was reverse transcribed using RevertAid cDNA synthesis kit following manufacturer’s instructions. qRT-PCR analysis was performed in Step One Real-time qPCR system (Applied Biosystems, USA). The SYBR green was used to run the PCR reactions of 10 μl each, containing 0.2 μl cDNA, 0.2 μM primers, 5 μl of SYBR Premix Taq (Takara, Otsu, Japan) and MQ water to make up the final volume. The thermo profile of reaction follows as: denaturation at 94 °C for 1 min, followed by 40 cycles each of denaturation for 10 s at 94 °C, annealing at 60 °C for 20 s and extension at 72 °C for 25 s. Actin gene was used as an internal control to assess the relative transcript levels of the genes. The relative quantification method (2^-ΔΔCT^) was used to analyse quantitative variation between the replicates [40]. Three replicates of each cDNA sample were taken for qRT-PCR analysis and repeated twice. Further, data were analyzed statistically.

**Table 2:** List of primers used in the study

S.No	Primer name	Primer Sequence (5′-3′)	Direction
1.	DXPR	5’CGGTGAACTTAAGGAGGC3’	Forward
2.	DXPR	5’TGGCTTCAAACCTGCACA3’	Reverse
3.	DPCMEK	5’GGATGGTTTTCATGATCTGGC3’	Forward
4.	DPCMEK	5’GCCTTTATGATCAAGTTTCG3’	Reverse
5.	MECDPS	5’GTGCTGCTTCACTGCGTG3’	Forward
6.	MECDPS	5’CTCGTGCATCAGTCGCAC3’	Reverse
7.	HMBEDPR	5′ ACATGTCCATGGGTGTCT3’	Forward
8.	HMBEDPR	5’CAAGTTTACCCCCAAGAATG3’	Reverse
9.	HMG	5′ TTCCTTCTGCGCCTAAAATGATG3’	Forward
10.	HMG	5’AATAGAGGCCGCACGAAAACAGT3’	Reverse
11.	MK	5’CTTGCTGTTGGAGTTTCAG3’	Forward
12.	MK	5’ATCTGACAAAGCAAGCAG3’	Reverse
13.	PMK	5’GGTTGCAATGACTTCTATTC3’	Forward
14.	PMK	5’AACTTCAGGCTTGCAACT3’	Reverse
15.	DPMD	5’GCATGTGCATTCATTGCTTC3’	Forward
16.	DPMD	5’AGAACCTTGCCTTGCTAT3’	Reverse
17.	SLS	5’ATGTCCACAATCCACTGG3’	Forward
18.	SLS	5’ATTTGTTCAGCAGGTCATCGT3’	Reverse
19.	STR	5’CAGCAAGTGAGCGAGCACATT3’	Forward
20	STR	5’AAGAGGAATACCTTGGGCTTG3’	Reverse
21.	Actin	5’ATGACATGGAGAAGATCTGGCATCA3’	Forward
22.	Actin	5’AGCCTGGATGGCAACATACATAGC3’	Reverse
23.	rolB	5’GCTCTTGCAGTGCTAGATTT3’	Forward
24.	rolB	5’GAAGGTGCAAGCTACCTCTC3’	Reverse

### Hairy root induction

Three *Agrobacterium* strains ATCC15834, LB9402 and A4 were tested for their transformation efficiencies in *N*. *nimmoniana*. Prior to transformation, bacterial strains were grown for 2 days on solid Yeast Mannitol Broth (YMB) at 28 ± 2 °C. From each strain single colony of bacteria was inoculated into 10 mL of liquid YMB medium containing an appropriate antibiotic and grown overnight at 28 °C with vigorous shaking (180 rpm). One milliliter of the overnight cultures were used to inoculate in 100 mL of YMB medium and incubated at 28 °C under shaking until reaching O.D. 600. Bacteria were harvested by centrifugation at 5,000 g for 10 min at room temperature. The supernatant was discarded and bacterial pellet was resuspended in 20 mL fresh YMB containing various concentrations of acetosyringone (50–300 μM). These concentrated cultures were used for infection of explant materials. The explants were kept in sterile petri dishes and pricked manually with sterile needle. The injured samples were dipped in *Agrobacterium rhizogenes* cultures and incubated for 30 min at 28 °C. The infected explants were dried and pre-incubated for co-cultivation at 28 ± 2 °C for 48 h on MS basal medium. After period of co-cultivation cultures were washed twice with sterile water and transferred to MS basal medium containing 500 mg/L cefotaxime for removing excess bacteria. Hairy roots started emerging after 3–4 weeks from the wounded sites of the explants. These were then transferred into hormone free MS1/2 liquid medium for further growth.

### DNA extraction and PCR analysis

Genomic DNA was extracted from transformed hairy roots as well as from wild roots of *N. nimmoniana* using Wizard® Genomic DNA Purification system, according to manufacturer’s protocol (Promega, Madison, USA). Absorbance of DNA samples was estimated by using NanoDrop® ND-1000 spectrophotometer (NanoDrop Technologies, Wilmington, DE, USA). DNA quality was determined by the optical density ratios at 260 nm/280 nm and by analysing on 0.8% agarose gel followed by examination of band integrity. The transgenic nature of hairy roots was verified by amplification of *rol* B gene using gene specific *rol*B primers Table 2. The PCR program were as follows: initial denaturation at 95 °C for 5 min; followed by 35 cycles for 30 s at 95 °C; annealing at 50 °C for 30 s; extension at 72 °C for 50 s with a final extension at 72 °C for 1 min. Twenty microliter of PCR reactions were carried out in a thermal cycler (Biorad, Hercules, CA) containing 100 ng DNA, 2.5 mM MgCl_2_, 200 μM dNTPs, 400 nM each of the forward and reverse primers, 10x Taq buffer containing (NH_4_)_2_SO_4_, and 1 unit of Taq DNA polymerase (Fermentas, Glen Burnie, MD). Amplified regions were examined on agarose gel electrophoresis.

## Results

### Experimental design and effects of the pathway inhibitors on CPT accumulation in *Nothapodytes nimmoniana*

**Fig. 2:**
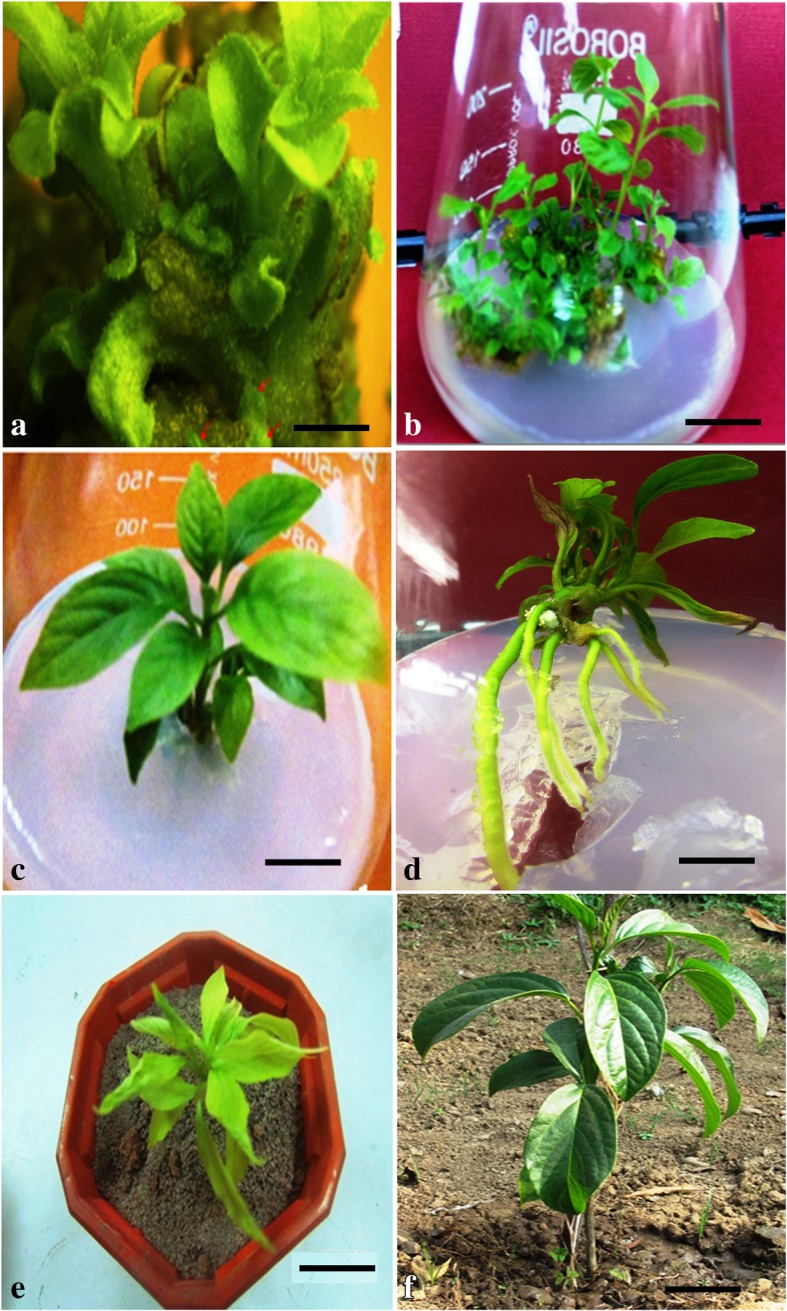
In vitro plant regeneration in *Nothapodytes nimmoniana*: Multiple shoot regeneration from nodal segments on Woody Plant Medium (WPM) supplemented with 9.08× 10^− 6^ M TDZ and 4.92 × 10^− 6^ M IBA (bar = 3 mm) (**a, b**); In vitro rooting of micro-shoots on Woody Plant Medium (WPM) supplemented with 14.7 × 10^− 6^ M IBA (bars = 40 mm and 30 mm) (**c, d**); Acclimatization in green house (bar = 4 cm) (**e**) and well established hardened plant under field conditions field (bar = 6 cm) (**f**)

**Fig. 3:**
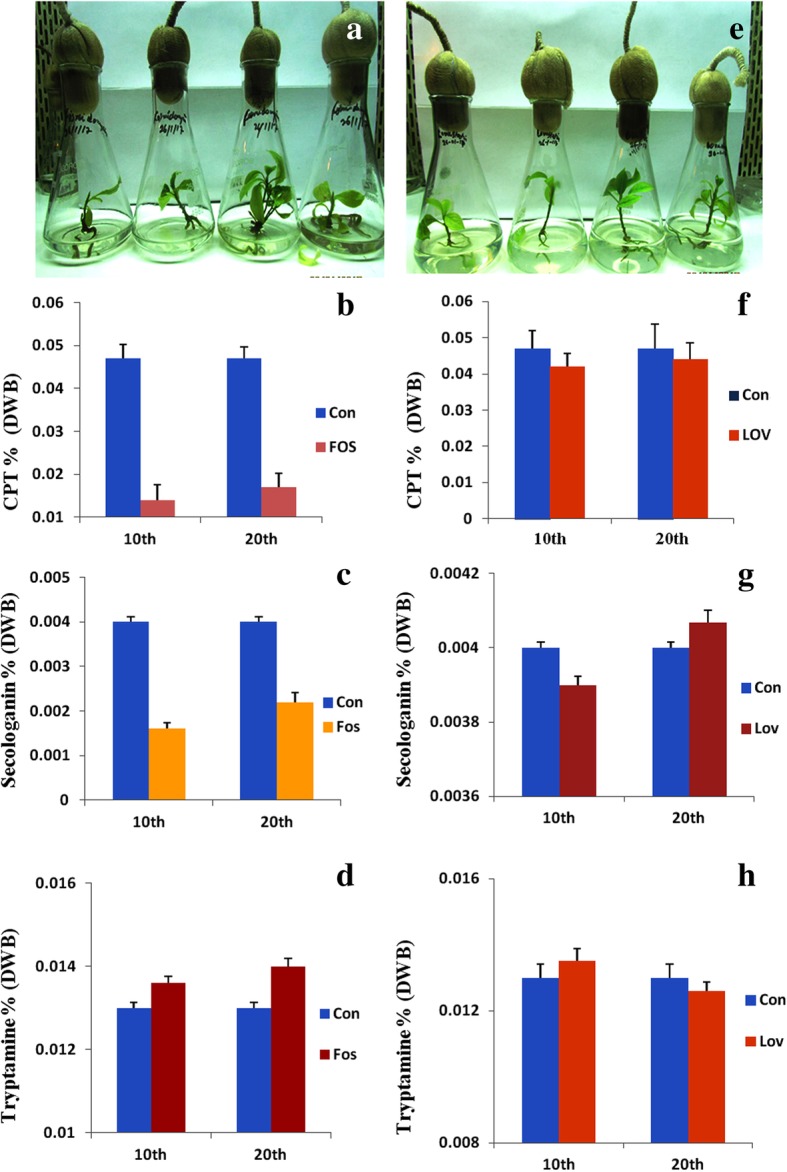
Effect of inhibitors on CPT accumulation: Rooted cultures of *Nothapodytes nimmoniana* grown in presence of fosmidomycin (150 μM) and HPLC demonstration of camptothecin (CPT), secologanin and tryptamine concentration (**a-d**); Lovastatin (100 μM) treated static cultures and HPLC analyses and quantification of camptothecin (CPT), secologanin and tryptamine concentration (**e-h**). Values are means with standard error

**Fig. 4:**
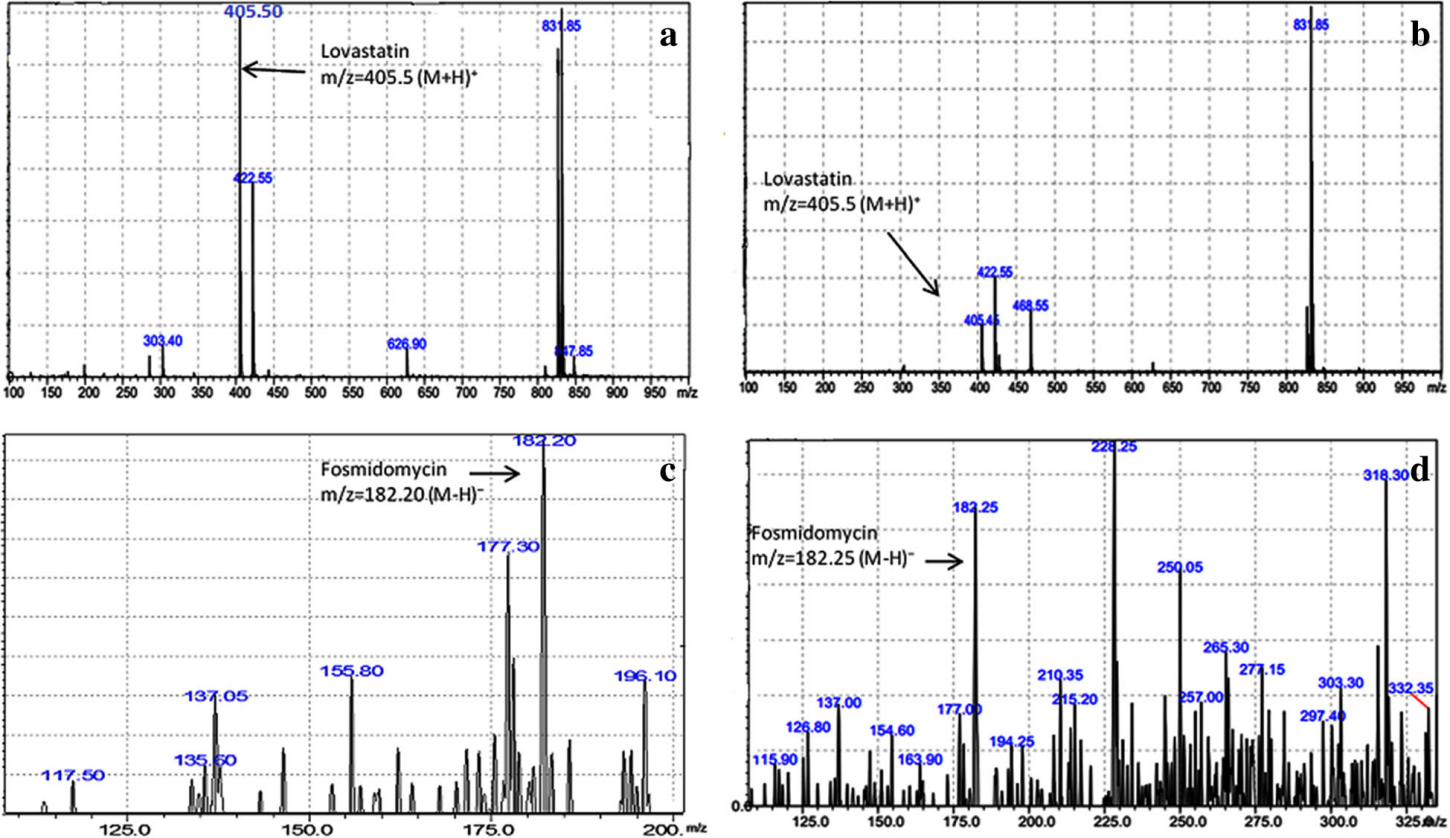
LC-MS/MS: Mass spectra of fosmidomycin (**a**); Presence of fosmidomycin in tissue extracts was confirmed by comparing mass spectra with the standard (**b**); mass spectra of lovastatin standard (**c**) and mass spectra of lovastatin present in plant extracts (**d**)

**Fig. 5:**
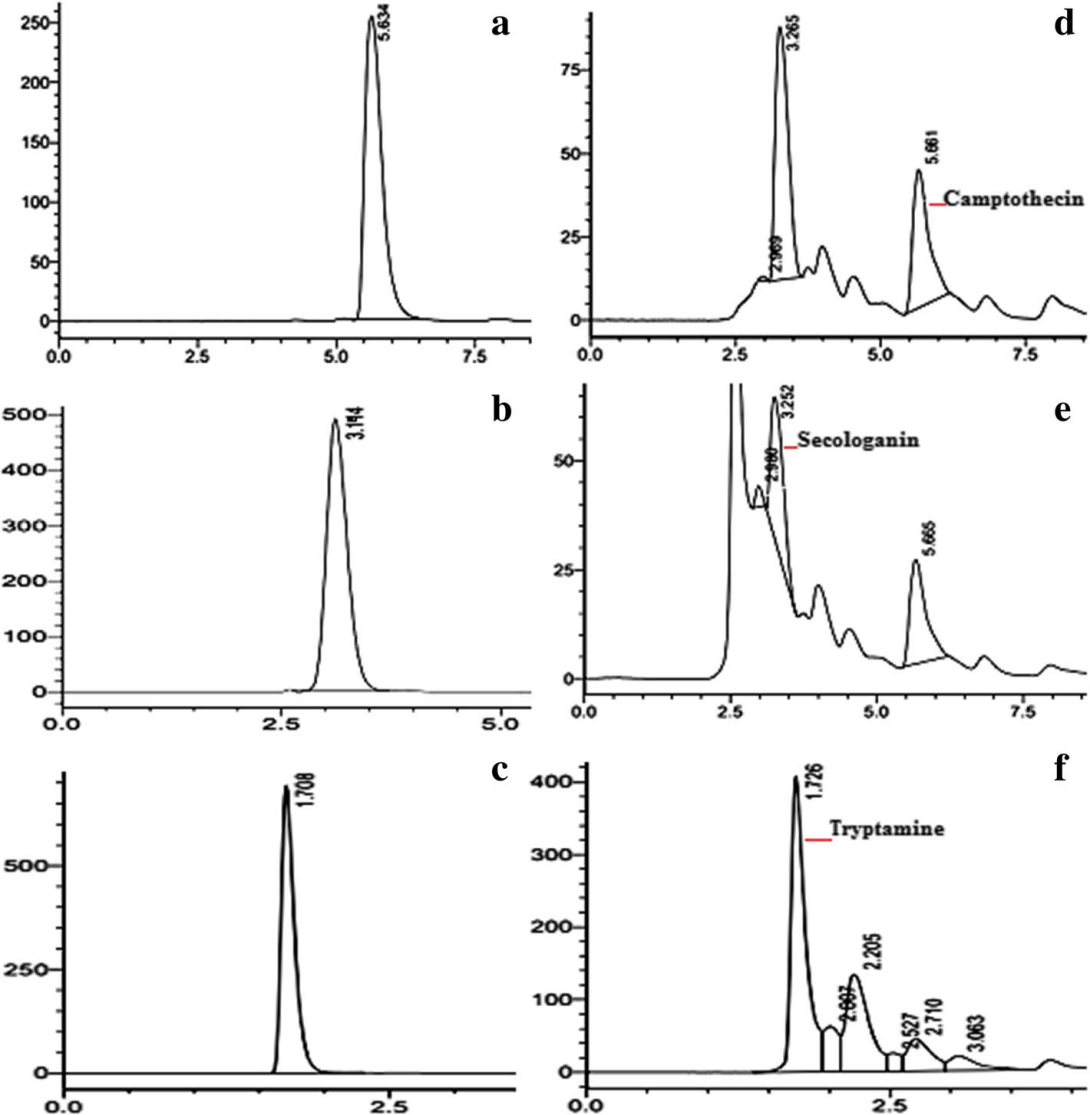
HPLC profiling: Chromatograms of authentic standards: camptothecin (**a**); secologanin (**b**) and tryptamine (**c**). HPLC profiles of tissue extracts for detection and quantification of seo-iridoid metabolites, camptothecin (**d**); secologanin (**e**) tryptamine (**f**). The HPLC conditions of secologanin and camptothecin are, acetonitrile: formic acid (99.5:0.5; v/v) delivered at a flow rate of 0.8 mL min^− 1^. The secologanin and camptothecin were eluted at retention time of 5.6 min and 3.2 min. Respectively. Mobile phase of tryptamine consisted of methanol: water: formic acid (50: 50: 0.1; v/v) at a flow rate of 0.9 mL/min. Tryptamine was eluted at a retention time of 1.7 min

From the nodal segments of field grown *N. nimmoniana,* an efficient and highly reproducible in vitro regeneration system was established on WPM fortified with different phytohormone concentrations and combinations (Fig. 2) and their morphogenetic response is summarized in the Table 1. Nodal explants exhibited maximum response in terms of multiple shoot induction on WPM supplemented with 9.08× 10^− 6^ M thidiazuron (TDZ) and 4.92 × 10^− 6^ M IBA with regeneration frequency of 65% and an average of 9.8 ± 0.60 shoots per explant. After 7–8 weeks of in vitro growth, fully developed elongated shoots were transferred to rooting WPM medium supplemented with 14.7 × 10^− 6^ M IBA. Further, in vitro regenerated rooted plantlets were employed to evaluate the flux perturbations caused by specific inhibitors of MEP and MVA pathways (Fig. 3a, e). These cultures were grown and acclimatized in liquid WPM for 2 weeks and subsequently grown in liquid WPM in presence of inhibitor fosmidomycin (150 μM) and lovastatin (100 μM). On 10th day LC-MS/MS scan of extracts treated with inhibitors showed the presence of fosmidomycin with calculated m/z _=_ 182.15 (M-H)^−^ and for lovastatin with calculated m/z_=_ 405.5 (M + H)^+^ (Fig. 4). The mass scan spectra of lovastatin standard, lovastatin in plant tissues, fosmidomycin standard and fosmidomycin in plant tissues are provided as Additional files 1, 2, 3 and 4, respectively. Further, green shoots harvested at 10th and 20th day of the treatment were investigated for chemical and qRT-PCR analyses. The HPLC profiles of authentic standards CPT, secologanin and tryptamine and their detection in the extracts are shown in Fig. 5. In presence of inhibitors, differential accumulation of CPT, secologanin and tryptamine were observed. Phytochemical analysis of cultures treated with fosmidomycin showed highly significant reduction in the metabolites of iridoid pathway like secologanin and CPT while the shikimate pathway metabolite tryptamine an immediate precursor of strictosidine showed only slight increase (Fig. 3b, c, d). The results revealed that on day 10 of fosmidomycin treatment there was about 57 and 71.5% reduction of secologanin and CPT content respectively, as compared to control. After the initial drop at 10th day, secologanin and CPT levels were recovered to ~ 17 and 7% respectively, in samples harvested on day 20 as compared to control (Fig. 3b, c). Moreover, tryptamine showed 4.6 and 7.69% increase in their content on day 10 and day 20 respectively (Fig. 3d). On the other hand, lovastatin was found to be least effective. The chemical analysis of lovastatin treated samples displayed a reduction of only 11 and 7% in CPT content at 10th and 20th day respectively (Fig. 3f). Secologanin content drops 7.5% at day 10 and comes to normal level at day 20 (Fig. 3g). Tryptamine content slightly increased upto 3.84% at day 10 and attained normal level at day 20 (Fig. 3h). Taken as a whole, major fluctuation in the accumulation of metabolites was observed in fosmidomycin treated samples while the effect of lovastatin was nearly negligible.

### Inhibitor mediated changes in the expression of MEP/MVA pathway genes

**Fig. 6:**
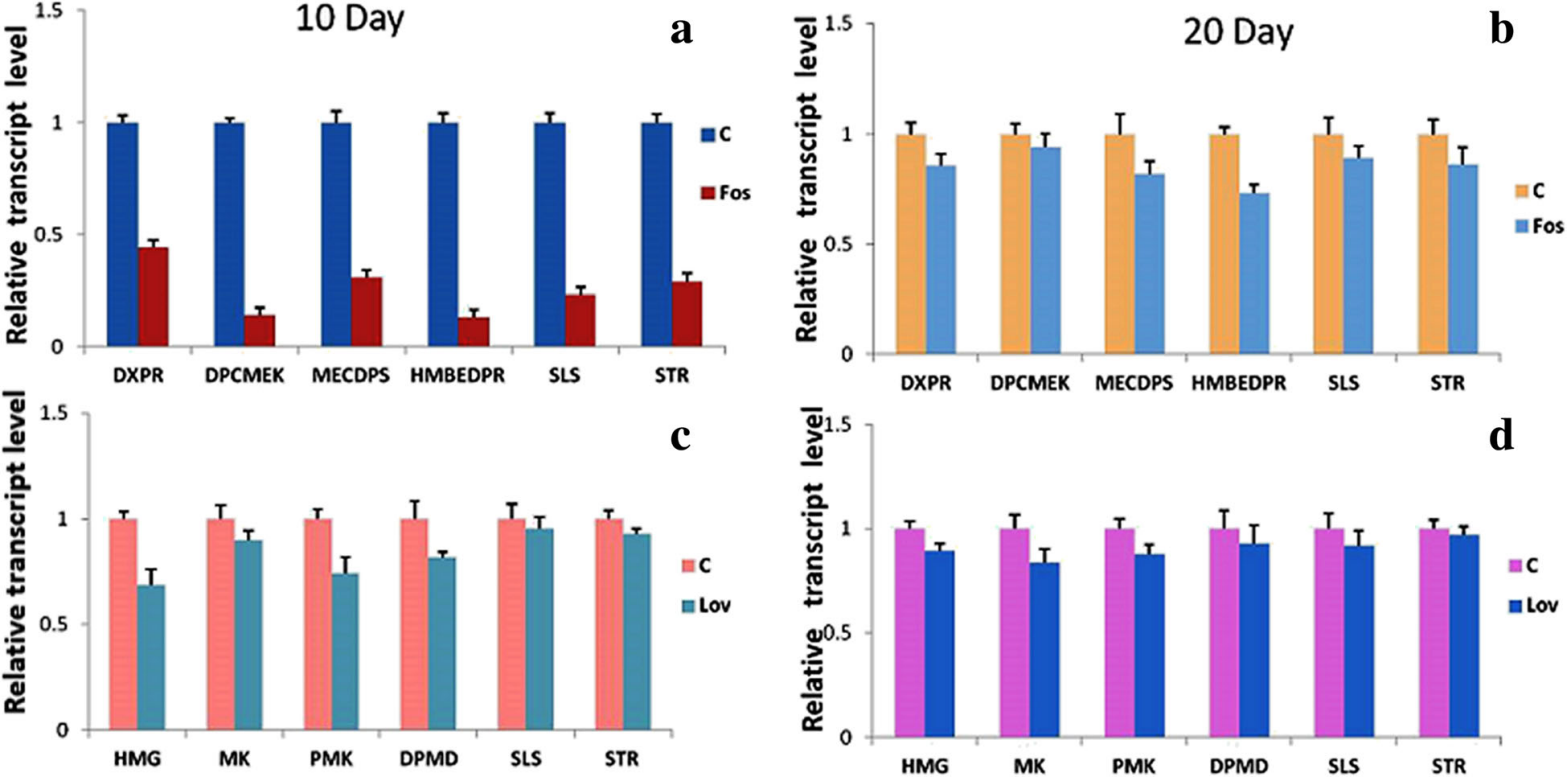
Real-time expression of DXR and HMG along with other downstream genes in fosmidomycin and lovastatin treated cultures: Quantitative estimation of the relative expression of DXR, DPCMEK, MECDPS, HMBEDPR, SLS, STR at day 10 and day 20 in presence of fosmidomycin (**a**, **b**). Expression of HMG, MK, PMK, DPMD, SLS, STR at day 10 and day 20 in presence of lovastatin (**c**, **d**). β-actin was used as an endogenous control to normalize the expression of genes. The data were compared and analyzed with one-way ANOVA using GraphPad Prism 6 software

To investigate the inhibition effects of fosmidomycin and lovastatin at transcriptional level, the expression levels of DXR and HMG were monitored by qRT-PCR. In presence of fosmidomycin, 0.57–0.15 fold reduction of DXR expression level was observed at 10th and 20th day of the treatment as compared to control (Fig. 6a, b). Similarly, lovastatin reduced the expression levels of HMG upto 0.32–0.11 fold (Fig. 6c, d). Furthermore, expression levels of three more downstream genes of MEP pathway (DPCMEK, MECDPS, HMBEDPR) and MVA pathway (MK, PMK, DPMD) were also examined (Fig. 6a-d). These genes also showed 0.87–0.10 fold reduction in their transcript levels. Additionally, key pathway genes SLS and STR of seco-iridoid pathway were also investigated which showed 0.71–0.14 fold reduction in their transcript levels in presence of fosmidomycin (Fig. 6a, b). In lovastatin treated cultures a slight decrease of 0.05–0.08 fold in SLS and STR transcript levels were observed as compared to control (Fig. 6c, d). Overall, the inhibitors fosmidomycin and lovastatin strongly reduced the transcript levels of DXR and HMG respectively. It was further obvious in terms of modulating the expression levels of other examined downstream genes viz. DPCMEK, MECDPS, HMBEDPR, MK, PMK, DPMD, SLS, STR.

### Hairy root induction and CPT quantification

**Fig. 7:**
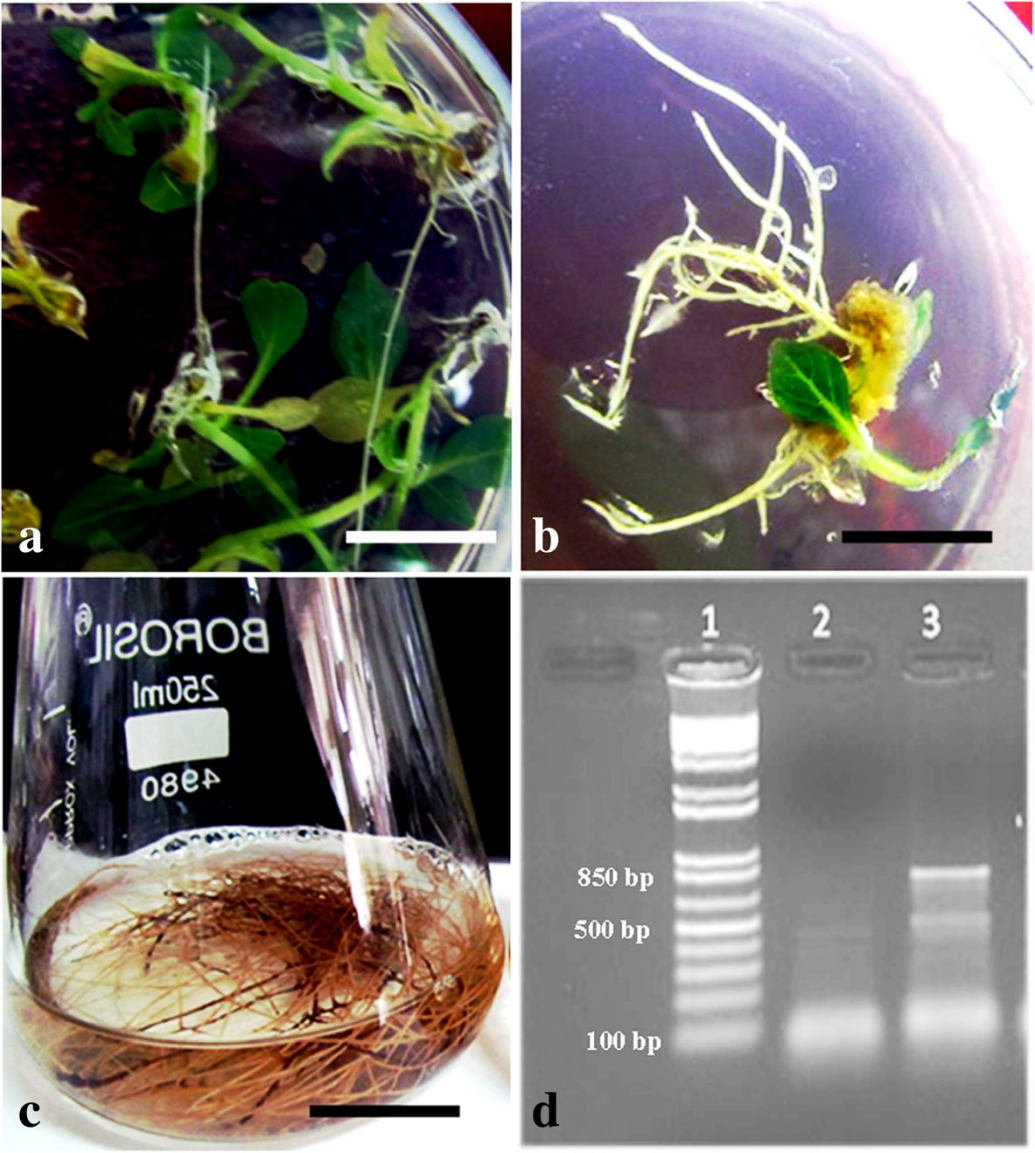
*Agrobacterium rhizogenes* induced hairy roots in *Nothapodytes nimmoniana*: Hairy roots grown on hormone free MS medium supplemented with 500 mg/L cefotaxime (bar = 10 mm) (**a, b**). Hairy roots grown in hormone free MS liquid medium supplemented with 3% sucrose (bar = 15 mm) (**c**); PCR amplification of *rol* b gene from genomic DNA of hairy roots (**d**)

**Fig. 8:**
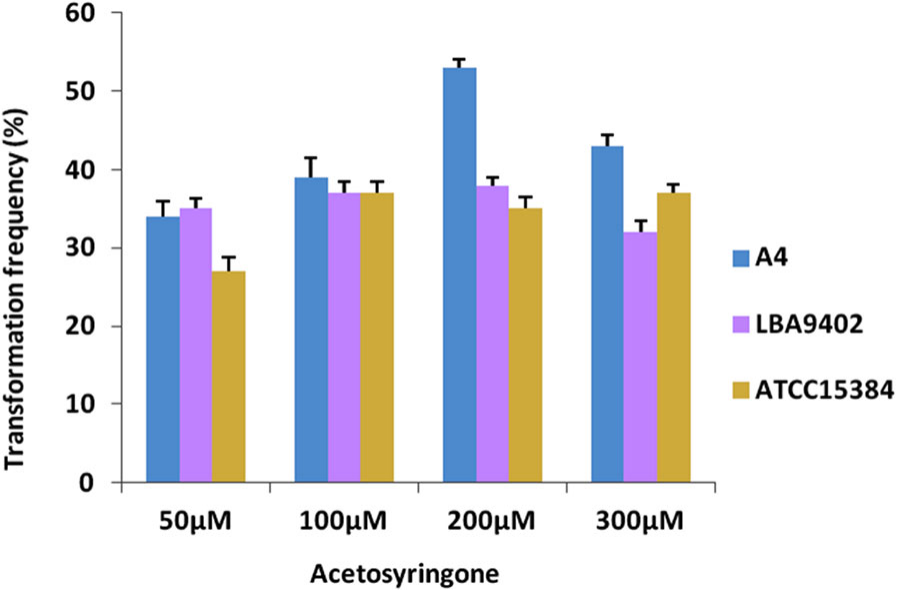
Effect of acetosyringone concentration on hairy root induction: Transformation frequency of *Agrobacterium rhizogenes* LBA9402, ATCC15834 and A4 strains at different acetosyringone concentrations. Error bars represent the mean of three replicates

**Fig. 9:**
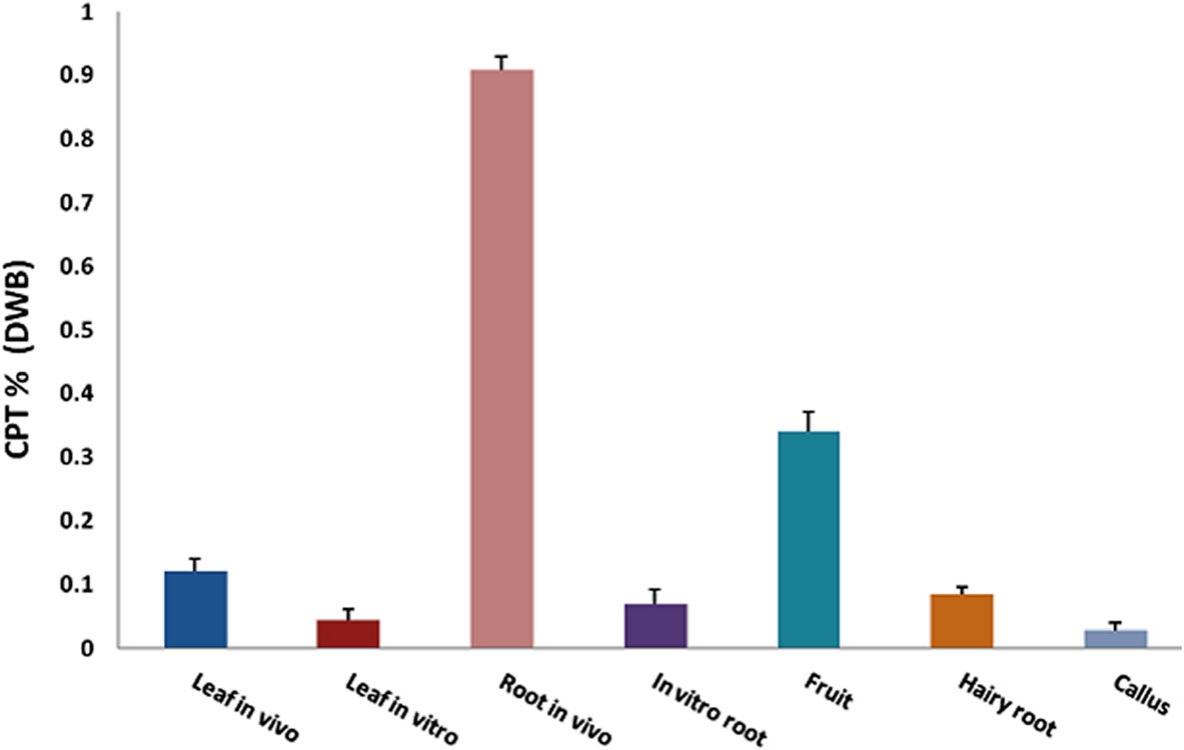
Comparative HPLC quantification of CPT content: Camptothecin content in different tissues of in vitro regenerated (leaf, root, callus, hairy root) and in vivo (leaf, root) plants. All the values obtained were means of triplicate with standard error

*Agrobacterium rhizogenes* mediated hairy root induction protocol was developed after optimizing several parameters that affect transformation (Fig. 7). Organogenetic shoots regenerated via intervening callus were taken as explants for hairy root induction. Three *Agrobacterium* strains viz. ATCC15834, LB9402 and A4 were tested for their effectiveness. All of the strains induced hairy roots successfully but their transformation efficiency was affected at different acetosyringone concentrations. At 200 μM acetosyringone concentration, A4 strain showed highest transformation frequency (53%) followed by LBA9402 (38%) and ATCC 15834 (35%) (Fig. 8). Transgenic nature of hairy roots was demonstrated by PCR amplification of *rol* B gene. A 780 bp fragment was amplified from genomic DNA isolated from hairy roots, whereas no such band was observed in untransformed aseptic roots taken as control. Hairy roots were further grown in hormone free half-strength MS liquid medium for 3 weeks and harvested for CPT analysis (Fig. 7d). For assessment of CPT content, methanolic extracts of in vitro (hairy root, shoot and callus) and in vivo (fruit, leaf and root) tissues were prepared for HPLC analysis. The amount of CPT on dry weight basis (DWB) in the fruit, leaf and root tissues of mother plant was 0.34, 0.12 and 0.9%, respectively. While as, in the in vitro regenerated callus, leaf tissues, root and hairy roots the CPT content was 0.02, 0.044, 0.069 and 0.08% respectively. Interestingly, CPT content of hairy roots (0.08%) was moderately higher in comparison to corresponding axenic in vitro roots (0.069%) (Fig. 9).

## Discussion

Plants produce an amazing diversity of low molecular weight chemical constituents that display a wide range of structural diversity and biological activities. Bioactive compounds tend to accumulate in very small quantities and their chemical synthesis is often complicated [14, 36]. However, in vitro culture systems offer a plausible tool for understanding the physiological, metabolic, biochemical and molecular regulation of plant secondary metabolism and are extensively employed for clonal propagation, as a gateway for metabolic engineering and provides a viable production platform for high value pharmaceutically important molecules [41]. In this context, an efficient in vitro regeneration system of *N. nimmoniana* was established on WPM supplemented with 9.08 × 10^− 6^ M TDZ and 4.92 × 10^− 6^ M IBA. In plants, biosynthesis of secondary compounds is exceedingly intricate and highly coordinated process as far as their biosynthesis, storage and temporal and spatial expression is concerned [42]. Invariably, the limited yield of potential bioactive constituents in plant tissues presents a challenge for large scale drug production [43]. Pathway engineering has galvanized the development of in vitro strategies in the form of plant cell and tissue cultures as viable optional production platforms for secondary metabolites that can be scaled up in controlled conditions [44]. Contextually, understanding of metabolic pathways is of tremendous importance for genetic manipulation to increase the target metabolite(s) as well as to alter the expression of key metabolic genes at molecular level [45, 46]. Despite the complex structure, MIAs are biosynthesized from the basic isoprene units i.e., isopentenyl pyrophosphate which in plants is produced via two different routes, the cytosolic MVA pathway and the plastidial MEP pathway [47]. However, studies have suggested a cross-talk of isoprene units in between these pathways [48]. These inferences have prompted intense research to decipher the components of cross-talk using feeding experiments. In *Salvia miltiorrhiza*, it has been reported that MVA pathway flux plays main role in cell growth, whereas the intermediates of MEP pathway are the major source of tanshinone biosynthesis [20]. Similarly, in *Picrorhiza kurroa* the role of the MEP pathway in picroside biosynthesis was determined by using enzyme inhibitors mevinolin and fosmidomycin along with aminooxy acetic acid, glyphosate and actinomycin D [31]. In *N. nimmoniana* camptothecin production involves a complex network of pathways including MEP, MVA, shikimate and seco-iridoid pathways. Present investigation is an attempt to diverge the precursor pool and to understand the role of MEP and MVA routes in camptothecin production. Using pathway specific inhibitors for flux perturbation creates a sound understanding of biosynthetic routes, their respective end products and paves way for effective biotechnological interventions. Taken together, it is plausibly essential to establish the major precursor contributing pathway toward the target metabolite involving the diversion of substrate flux. In the present study, two specific inhibitors fosmidomycin and lovastatin were selected for inhibiting two important enzymes DXR and HMG of the MEP and MVA pathway respectively and their inhibition concentrations were tested as reported previously [31]. Chemical analysis of cultures treated with fosmidomycin showed a reduction of 64–71.5 and 40–57% in CPT and secologanin accumulation respectively. Furthermore, shikimate pathway product tryptamine content increased slightly in the range 4.61–7.69%. This might be due to strong reduction of secologanin content which condenses with tryptamine to produce strictosidine, a universal precursor of MIAs. Moreover, CPT and secologanin concentrations were not reduced significantly in presence of lovastatin and the tryptamine content remained nearly unaltered. Thus, the ability to contribute for CPT accumulation appears to be more effective via plastidial pathway than through cytosolic pathway. It is an ample indication of the predominant role of MEP pathway in CPT biosynthesis as evident from the empirical experimental results. These results are tenable and in accord with the previous findings related to pathway inhibition studies in *Catharanthus roseus*, *P. kurrooa* and *S. miltiorrhiza* [20, 31, 48]. Moreover, lovastatin treatment showed a small decrease in CPT content that might be due to the lack of metabolite exchange between two compartmentalized pathways. Oliver et al. [49] also reported that there is transient reduction of sterol levels by inhibition of HMGR enzyme of MVA pathway. This finding revealed that MVA pathway may contribute for the lack of cytosolic isopentenyl pyrophosphate needed for the synthesis of cytosolic sterols [32]. Such a cross-talk between cytosolic and plastidial pathways has also been discussed in *S. miltiorrhiza* [20]. Furthermore, present investigation reveals that both the inhibitors reduce secologanin and CPT content which prompted us to examine their effect at transcriptional level. Our qRT-PCR analysis of MEP and MVA and seco-iridoid pathway genes showed an altered gene expression in presence of inhibitors. The expression of DXR and HMG along with other downstream genes (DPCMEK, MECDPS, HMBEDPR, MK, PMK, DPMD, SLS, STR) showed subdued expression levels as compared to control. This strongly suggests the inhibition of DXR and HMG enzymes possibly leading to decrease in downstream intermediates of MIA pathway. Further, these changes are apparent at transcription level. From previous studies, it has been expounded that the posttranscriptional control may play an important role in the expression and regulation of genes.

*Agrobacterium rhizogenes* mediated hairy root induction is considered as robust alternative to produce high value secondary metabolites and fundamental research tool for understanding the biosynthetic pathways via genetic manipulation of committed steps [50]. An example of a successful attempt of metabolic engineering concerning with MIA biosynthesis strategy is the simultaneous and separate overexpression of geraniol 10-hydroxylase (G10-H) and strictosidine synthase (STR) in *Ophiorrhiza pumila* hairy roots. The co-overexpression of G10-H and STR genes in transgenic lines of *O. pumila* has enhanced about 56% of CPT accumulation as compared to control line and single gene transgenic lines [51]. In the present study, hairy roots were induced via three *Agrobacterium* strains (LBA9402, ATCC15834 and A4) and their transformation efficiency was assessed. A4 strain showed the maximum transformation efficiency, whereas, the strains LB9402 and ATCC15834 were comparatively less effective in rhizogenesis response. The phenolic compound acetosyringone enhances virulence of *Agrobacterium* strains and its concentrations have been reported to modulate the frequency of transformation [52]. In *Berberis aristata* 100 μM acetosyringone concentration was found to be more effective whereas, 200 μM concentration in *P. kurrooa* [53, 54]. To evaluate the effect of acetosyringone in transformation frequency in the present study, different concentrations (50–300 μM) were tested and among these 200 μM was found to be the most effective. Secondary metabolite accumulation fluctuates in different environmental conditions [55] and a number of environmental factors such as light, temperature and humidity influence biochemical pathways [56]. In *C. accuminata* cell specific expression analysis of tryptophan decarboxylase (*Ca*-TDC1) and 10-hydroxygeraniol oxidoreductase (*Ca*-HGO) has revealed that both *Ca*-TDC1 and *Ca*-HGO were expressed in leaf and stem, but their expression was not observed in root. These results demonstrate that root is presumably the accumulation site of CPT [57]. In the present study chemical analysis of *N. nimmoniana* revealed higher accumulation of CPT in root as compared to leaf. Moreover, in *C. accuminata* CPT quantification from in vitro roots regenerated from microcuttings showed lower accumulation of CPT than that of in vivo roots [58]. However, HPLC analysis of in vitro regenerated *O. mungos* showed comparable amount of CPT with the in vivo grown plants [59]. In *N. nimmoniana* chemical analysis of in vivo and in vitro tissues showed differential CPT accumulation. Low content of CPT was observed in in-vitro regenerated hairy roots, calli, leaf and root tissues in comparison to in vivo plant tissues. Moreover, calli and suspension cultures of *C. accuminata* failed to produce CPT which is in contrast to calli of *N. nimmoniana* [60]. Rapidly growing and well established hairy roots of *O. pumila* produce CPT up to 0.1% on dry weight basis and hairy roots of *C. accuminata* produce and secrete camptothecin (1.0 mg/g dry weight) and 10-hydroxycamptothecin (0.15 mg/g dry weight) into the medium at concentrations equal to or greater than normal roots of *C. accuminata* [61, 62]. In the present investigation, HPLC analysis of hairy roots regenerated from in vitro leaves of *N. nimmoniana* showed 0.08% of CPT content which is relatively higher than the untransformed aseptic roots (0.069) obtained from in vitro cultures and slightly lower than the observed content in *C. accuminata* and *O. pumila* [61, 62].

## Limitations

Due to complex structure of camptothecin molecule, its industrial biosynthesis is prohibitive and untenable. Moreover, it is produced in low concentrations and its pathway modulation is hindered due to unresolved biosynthetic machinery. Therefore, exploring the pathway precursor flux diversion for metabolic intensification may plausibly pave way for enhanced *in planta* production via biotechnological interventions. Differential accumulation of CPT in different biological resources such as hairy roots, developmental tissues, callus, leaves, roots etc. of *N. nimmoniana* can facilitate pathway resolution through system biology approach involving transcriptomics, genomics, proteomics and metabolomics to generate correlates of genes and metabolites in a given biological system. In future, this integrated ‘omics’ approach can facilitate the prediction of unidentified and novel genes and their enzymes involved in hitherto unresolved biosynthesis of camptothecin.

## Conclusions

To refine the effect of inhibitors on pathway flux perturbations towards isoprenoid end-product CPT, the inhibitor mediated changes were monitored at metabolite level via HPLC as well as at transcriptional level by qRT-PCR. In presence of specific enzyme inhibitors the differential accumulation of CPT was observed and these changes were also reflected at transcriptional level. Present investigation established that MEP pathway is the major route for providing monoterpene moiety to CPT biosynthesis. Additionally, to conduct inhibitor studies in relation to CPT production, an efficient in vitro regeneration system was developed as an experimental system. We also successfully induced transformed hairy root cultures employing *Agrobacterium rhizogenes*. Currently, hairy roots are being up-scaled for further MVA pathway intensification via genetic engineering.

## Additional files

Additional file 1: LC/MS mass scan of lovastatin standard. (PDF 65 kb)

Additional file 2: LC/MS mass scan of lovastatin treated plant tissue. (PDF 65 kb)

Additional file 3: LC/MS mass scan of fosmidomycin standard. (PDF 271 kb)

Additional file 4: LC/MS mass scan fosmidomycin treated plant tissue. (PDF 65 kb)

## Data Availability

All data generated or analysed during this study are included in this published article and in supplementary information files.

## Abbreviations

DPCMEK: Diphosphocytidyl-2-methyl-D-erythritol kinase
DPMD: Diphosphomevalonate decarboxylase
DXR: Deoxy-xylulose-5-phosphate reductoisomerase
HMBEDPR: Hydroxy-3-methyl but-2-enyl diphosphate reductase
HMG: Hydroxymethylglutaryl-CoA reductase
MECDPS: Methyl-D-erythritol 2,4-cyclodiphosphate synthase
MEP: Methylerythritol phosphate
MEPCT: Methyl erythritol-4-phosphate cytidyltransferase
MK: Mevalonate kinase
MVA: Mevalonate
PMK: Phosphomevalonate kinase
SLS: Secologanin synthase
STR: Strictosidine synthase
