# Supplementary material for: Metabolic and transcriptional analyses in response to potent inhibitors establish MEP pathway as major route for camptothecin biosynthesis in Nothapodytes nimmoniana (Graham) Mabb
Source: BMC Plant Biol. 2019 Jul 10;19:301. doi: 10.1186/s12870-019-1912-x (PMC6617690; doi:10.1186/s12870-019-1912-x)

SHIMADZU LabSolutions

Analysis Report

<Sample Information>

Sample Name	: LOV.		
Sample ID	:		
Data Filename	: 13-11-18 MASS SCAN6.lcd		
Method Filename	: MS Scan.lcm		
Batch Filename	: 13-11-18 MASS SCAN.lcb		
Vial #	: 1-50	Sample Type	: Unknown
Injection Volume	: 2 uL		
Date Acquired	: 13-11-2018 11:45:15	Acquired by	: System Administrator
Date Processed	: 13-11-2018 11:46:16	Processed by	: System Administrator

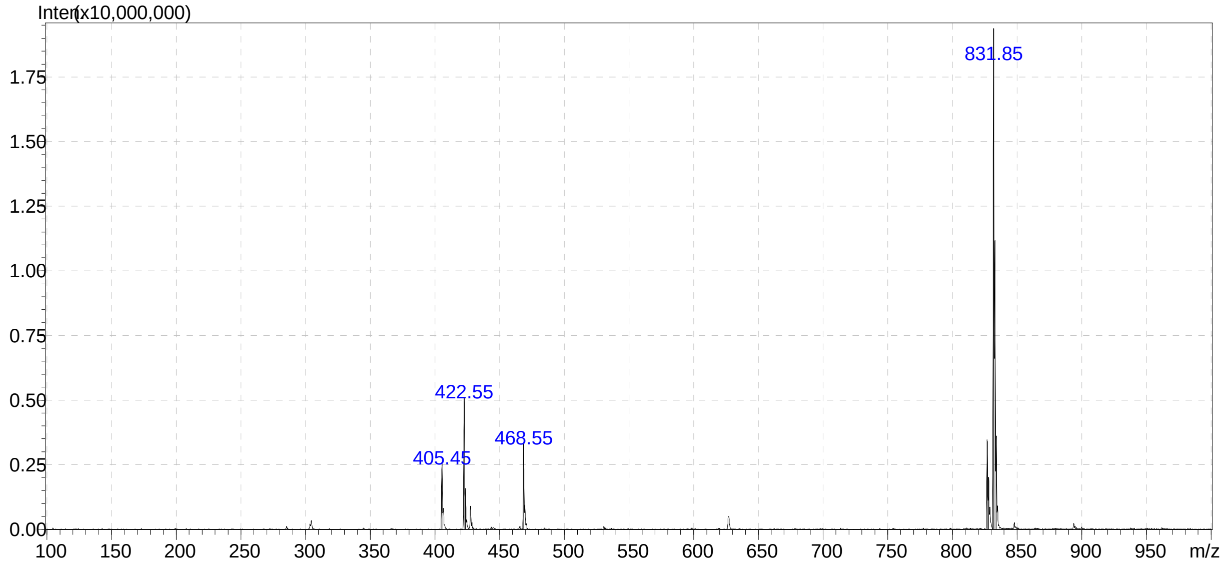

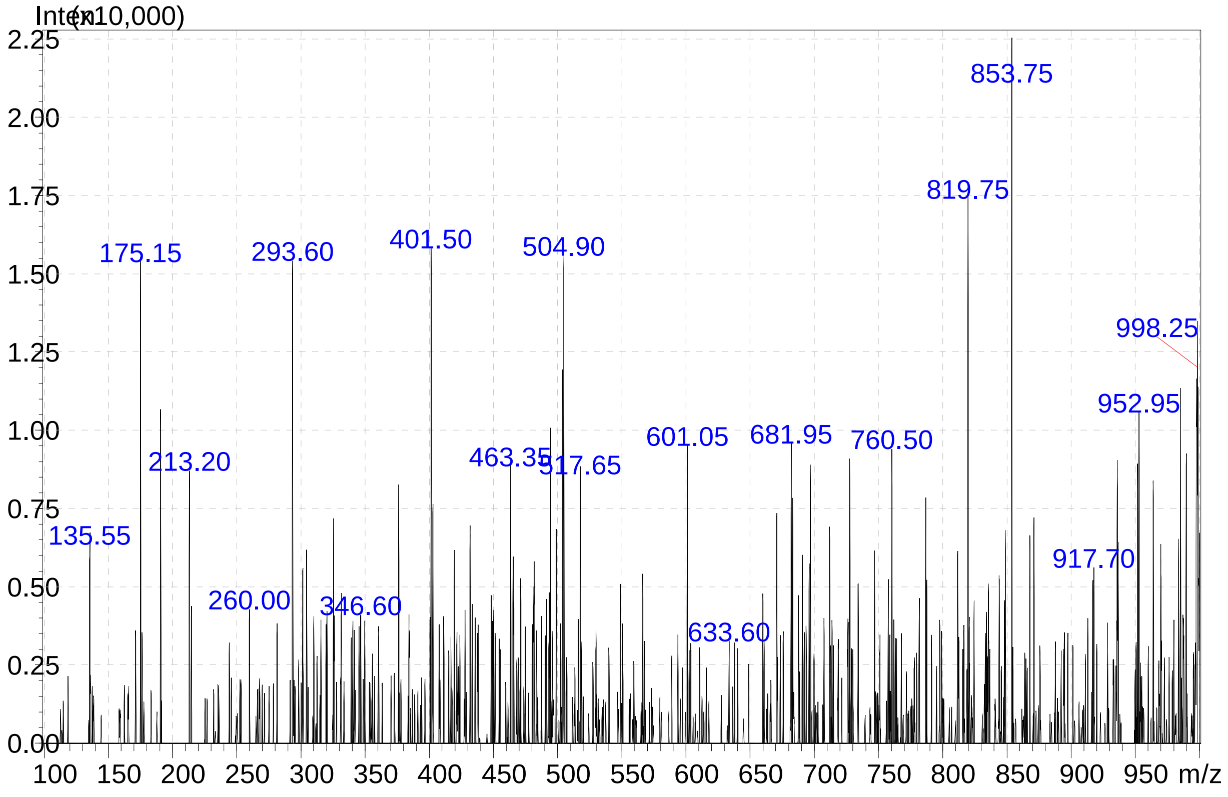

Supplement: Supplementary file 2 — LC/MS mass scan of lovastatin treated plant tissue. (PDF 65 kb) [file 12870_2019_1912_MOESM2_ESM.pdf]
