# Supplementary material for: Metabolic and transcriptional analyses in response to potent inhibitors establish MEP pathway as major route for camptothecin biosynthesis in Nothapodytes nimmoniana (Graham) Mabb
Source: BMC Plant Biol. 2019 Jul 10;19:301. doi: 10.1186/s12870-019-1912-x (PMC6617690; doi:10.1186/s12870-019-1912-x)

SHIMADZU LabSolutions

Analysis Report

<Sample Information>

Sample Name : FOS.
Sample ID :
Data Filename : 13-11-18 MASS SCAN4.lcd
Method Filename : MS Scan.lcm
Batch Filename : 13-11-18 MASS SCAN.lcb
Vial # : 1-48
Sample Type : Unknown
Injection Volume : 2 uL
Date Acquired : 13-11-2018 11:42:11
Acquired by : System Administrator
Date Processed : 13-11-2018 11:43:12
Processed by : System Administrator

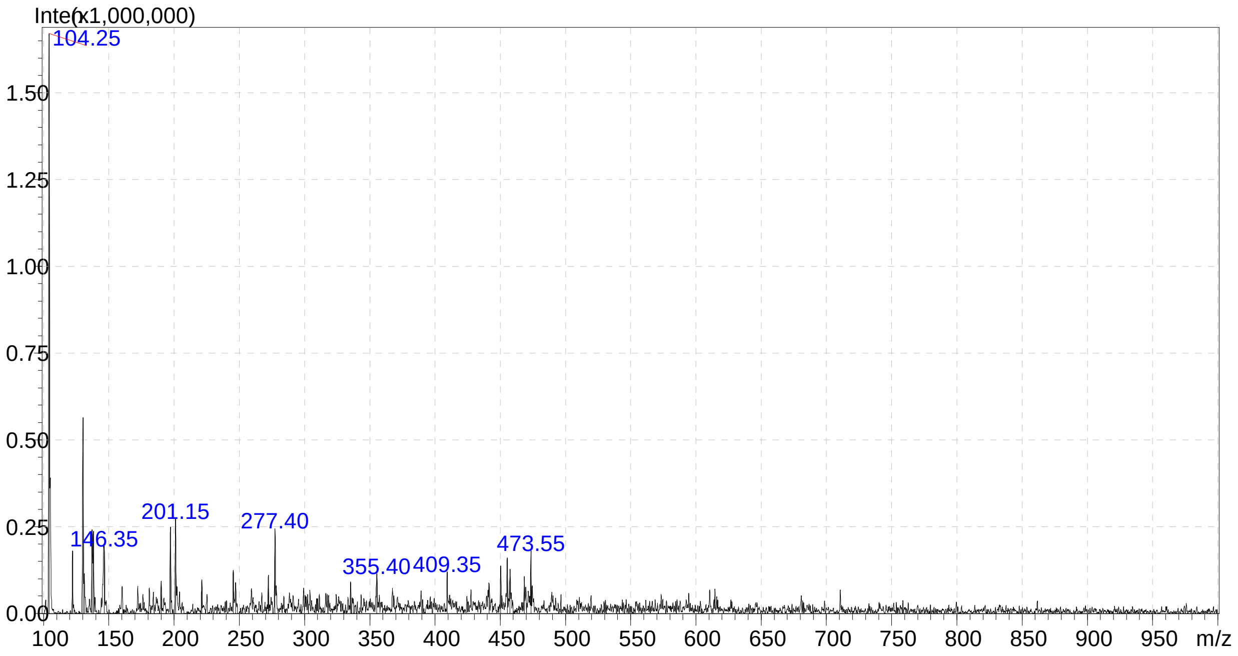

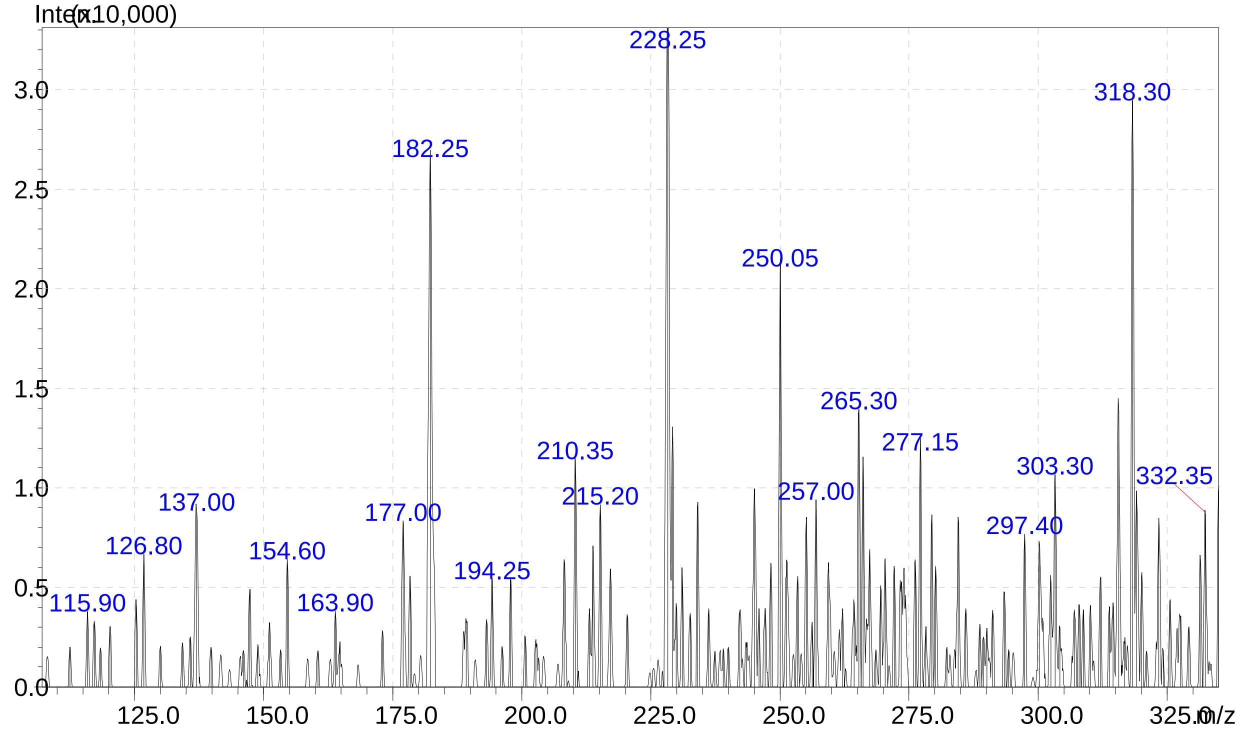

Supplement: Supplementary file 4 — LC/MS mass scan fosmidomycin treated plant tissue. (PDF 65 kb) [file 12870_2019_1912_MOESM4_ESM.pdf]
